# Descending aortic translocation for aerodigestive vascular compression

**DOI:** 10.1016/j.xjon.2026.101888

**Published:** 2026-06-05

**Authors:** Sarah Tsou, Peter Chiu, Ryan Benavente, Daniel Castellanos, Jennifer Brown, Lianne Cole, Benjamin Zendejas, Christopher W. Baird

**Affiliations:** a Department of Cardiac Surgery, Boston Children's Hospital, Boston, Mass; b Department of Cardiology, Boston Children's Hospital, Boston, Mass; c Department of Surgery, Boston Children's Hospital, Boston, Mass

**Keywords:** aerodigestive compression, congenital cardiac repair, congenital heart disease, vascular ring

## Abstract

**Objective:**

Patients with complex vascular ring anomalies can present with symptoms of esophageal and airway compression, occasionally persisting despite relief of the vascular ring, due to residual compression from the aorta. We report our early experience with descending aortic translocation to address aerodigestive vascular compression.

**Methods:**

This is a retrospective single-center review of patients undergoing descending aortic translocation. The operation involves dividing the distal aortic arch on cardiopulmonary bypass, relocating the descending aorta beneath the carina and right pulmonary artery, and anastomosis to the posterior aspect of the ascending aorta, thereby entirely relieving compression on the aerodigestive tract. Concomitant esophageal mobilization and tracheopexies are performed as needed.

**Results:**

From 2020 to 2023, 15 patients underwent descending aortic translocation. Anatomic variants included hairpin aorta (n = 2), circumflex aorta (n = 4), midline descending aorta (n = 7), and bowstring aorta (n = 2). Median age was 2 years. Preoperatively, most patients (80%) had mixed esophageal and airway symptoms and had undergone at least 1 prior operation (60%). Intraoperative bronchoscopy revealed improved airway patency for all patients. Postoperatively, 93% of patients had relief of respiratory symptoms, and 79% of patients had improved dysphagia. Postoperative complications included neurologic event (n = 1) with full resolution, right pulmonary artery compression requiring stenting (n = 1), and need for reoperation (n = 2).

**Conclusions:**

Descending aortic translocation is a novel approach that can provide symptomatic relief of aerodigestive compression related to complex aortic anomalies. For patients with persistent symptoms after initial vascular ring repair, ongoing aortic compression may need to be addressed, thus also raising the question of whether descending aortic translocation should be considered as a first-line therapy.

## Graphical Abstract

Retrospective single-center review (2020-2023) investigating the outcomes of descending aortic translocation, a novel approach for relief of aerodigestive compression related to complex aortic anomalies.

**Figure:**
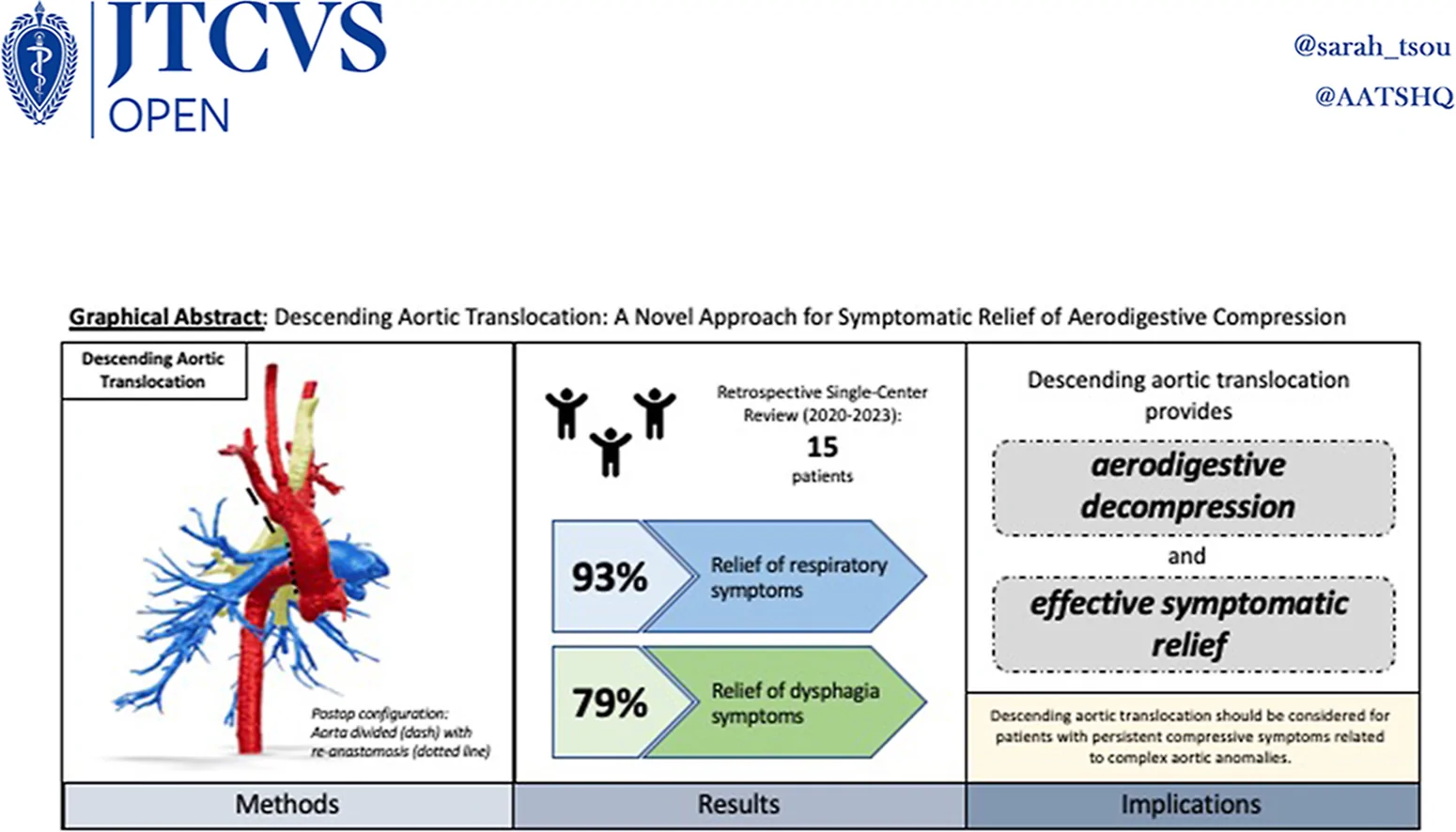

**Figure:**
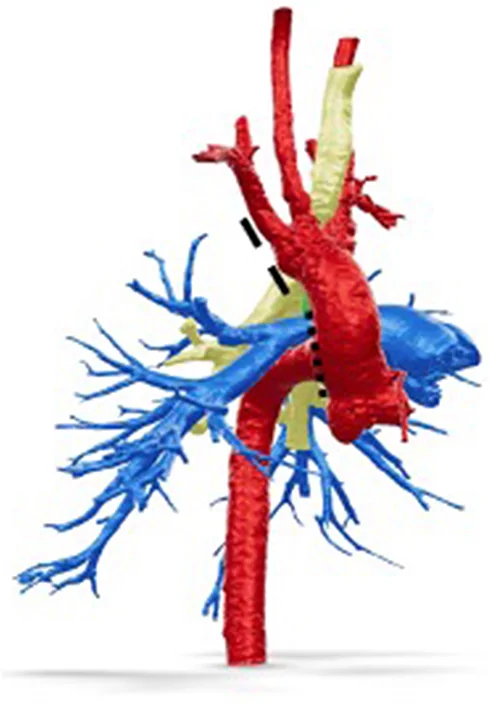
Descending aortic translocation, postoperative configuration. Aorta divided with reanastomosis.

**Central Message**

Aerodigestive symptoms may persist after vascular ring repair due to aortic compression. Descending aortic translocation is a novel operative approach that relieves this compression for symptom relief.

**Perspective**

Aerodigestive compression from complex aortic anomalies may persist despite standard repairs. This study highlights our early experience with descending aortic translocation, a novel operative technique that entirely relieves aortic compression on the aerodigestive tract, thereby offering durable symptom relief for the vast majority (93%) of patients.

Aerodigestive vascular compression may be due to congenital lesions of the aortic arch and its branches, anatomic variations in the relationship between the aortic arch and descending thoracic aorta, or acquired lesions after aortic reconstruction.^1^, ^2^, ^3^, ^4^, ^5^, ^6^, ^7^, ^8^ Patients typically present with symptoms related to extrinsic aerodigestive compression, including respiratory symptoms such as cough, stridor, frequent respiratory infections, and respiratory distress, as well as gastrointestinal symptoms such as dysphagia and failure to thrive.^9^ Although the standard surgical management techniques—including division of the vascular ring, subclavian-to-carotid transposition, aortopexy, or posterior tracheobronchopexy—are frequently sufficient for relief of symptoms, patients with aerodigestive vascular compression related to aortic position (ie, circumflex, hairpin, or midline aorta), narrow anteroposterior mediastinal space, or secondary to aortic reconstruction with inadequate space beneath the reconstructed arch may not have relief of symptoms.

Descending aortic translocation was first described by McKenzie and colleagues^10^ to address midline descending aorta in 5 patients with signs and symptoms of aerodigestive compression. Since then, multiple additional groups have reported successful relief of vascular-related aerodigestive compression for patients with not only midline descending aorta but also circumflex aorta and after aortic arch reconstruction with resultant left mainstem bronchial compression.^11^, ^12^, ^13^, ^14^ We report our experience with descending aortic translocation to treat vascular-related aerodigestive compression.

## Materials and Methods

This is a single-center retrospective cohort study of patients who underwent descending aortic translocation at Boston Children's Hospital between 2020 and 2023. The Institutional Review Board of the Boston Children's Hospital approved the study protocol and publication of data (IRB-P00045027, approved 3/20/2023). The requirement for informed consent was waived due to the retrospective nature of the study. The departmental database was interrogated, and data were abstracted from the medical record.

### Outcomes

The primary outcome of interest was achievement of symptomatic relief based on presenting symptoms. Secondary outcomes of interest included death, stroke, reintervention, chylothorax, and new vocal fold dysfunction. Reintervention included surgical, catheter-based, or endoscopic reintervention. Chylothorax was defined as persistently elevated chest tube output with confirmatory pleural triglyceride evaluation. Vocal fold dysfunction was defined as new vocal fold hypomobility or paralysis observed on postoperative awake flexible nasolaryngoscopy or laryngeal ultrasound. Notably, vocal fold movement evaluation is routinely performed preoperatively and postoperatively as part of our comprehensive approach to the management of vascular rings and aerodigestive vascular compression.^15^

### Preoperative Assessment

Before proceeding with descending aortic translocation, patients underwent a comprehensive preoperative multidisciplinary evaluation by teams from congenital heart surgery, pediatric surgery, cardiology, gastroenterology, otolaryngology, pulmonology, as well as social work and speech pathology when indicated. All cases were reviewed in a multidisciplinary vascular ring and airway compression conference to formulate a management plan.

Preoperative cardiac evaluation typically included computed tomography (CT) angiography and echocardiogram. According to CT angiography, patients with hairpin aorta (Figure 1, defined as a sharply bending aortic configuration involving a high-apex aortic arch, anterior-posterior narrowing, and ascending/descending aortic limbs aligned with one another on the right^16^); circumflex aorta (Figure 2); midline descending thoracic aorta (Figure 3); or with limited space underneath the reconstructed aortic arch—a phenomenon we have termed “bowstring aorta” (Figure 4)—were considered candidates for descending aortic translocation. Echocardiography was performed to rule out comorbid cardiac lesions including residual lesions from prior operations.

**Figure 1:**
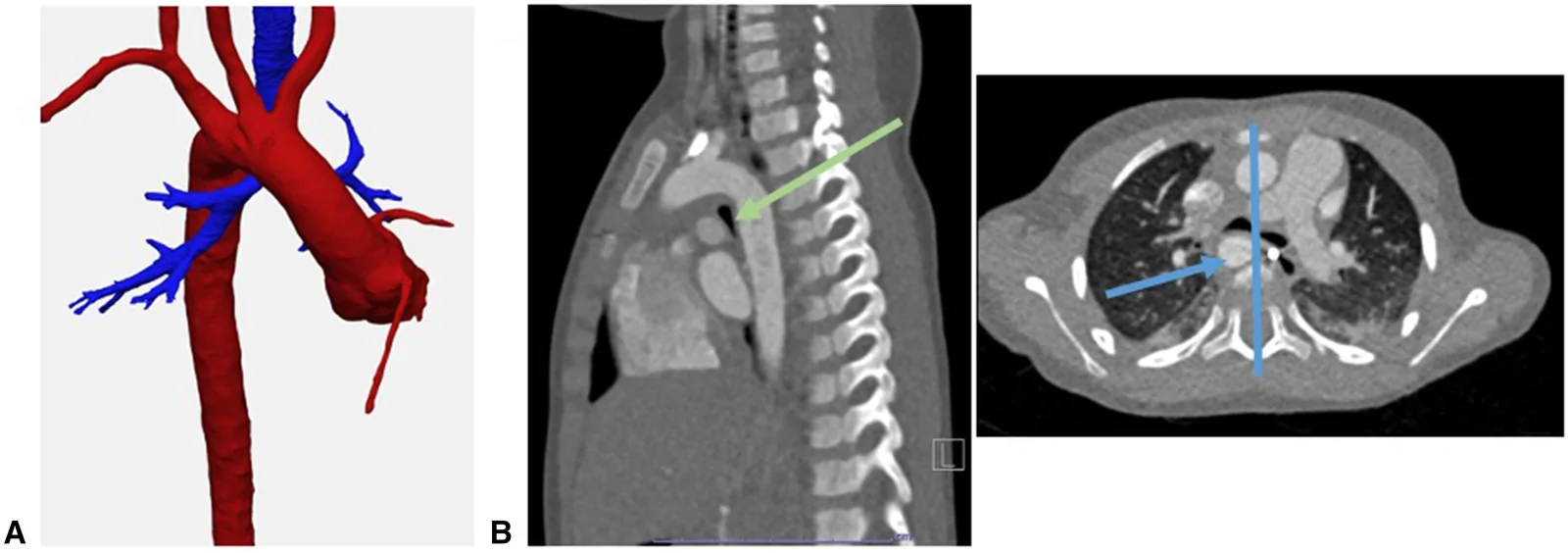
Hairpin aorta: (A) 3-dimensional (*3D*) reconstructed model and (B) sagittal and axial views from preoperative CT scan demonstrating an example of the hairpin aorta anatomic variant, classically with right-sided arch and right-sided descending aorta (*blue arrow*) leading to a tight “hairpin” configuration leaving little space between the aortic limbs (*blue line signifying midline*). The aberrant location of the descending aorta can lead to compression of the airway (*green arrow*).

**Figure 2:**
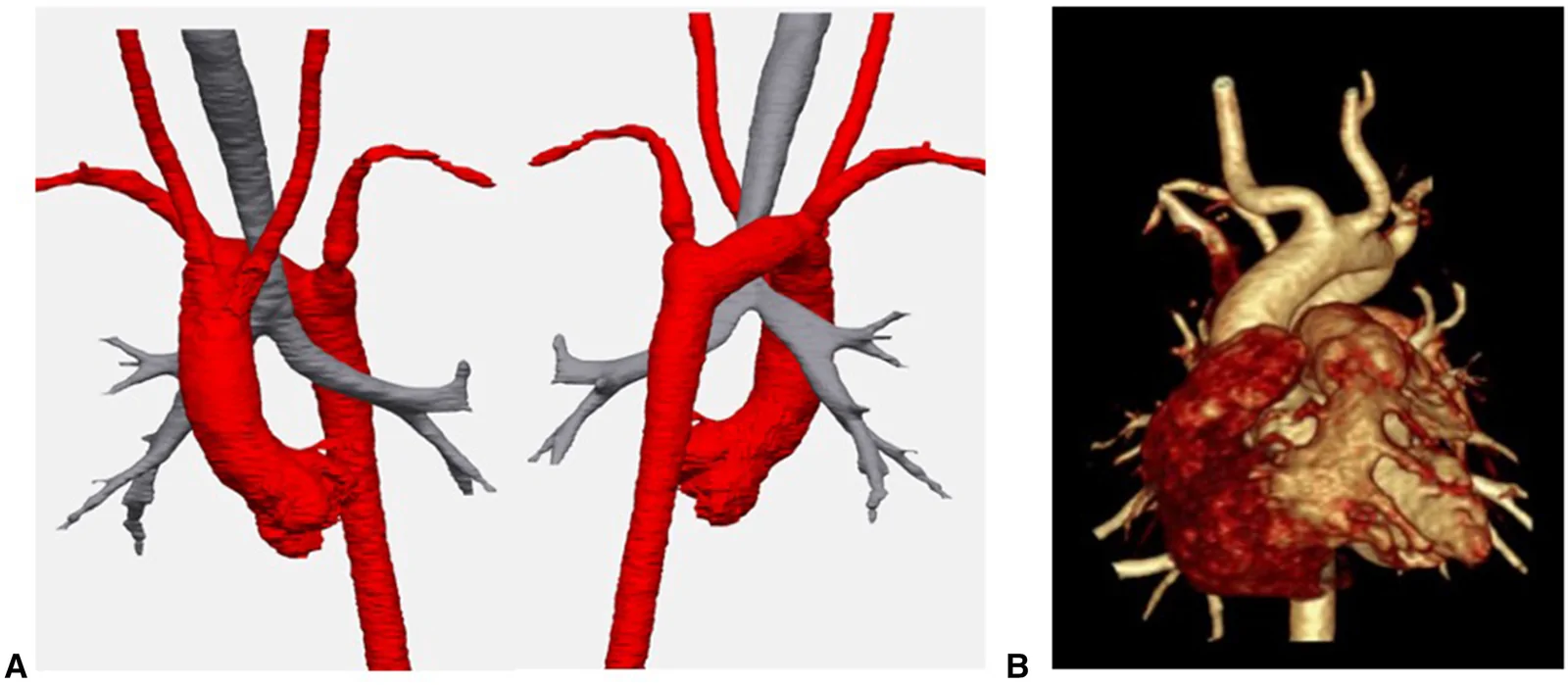
Circumflex aorta: (A) 3D reconstructed models of anterior-posterior and posterior-anterior views; (B) additional 3D reconstruction, captured from a preoperative CT scan, demonstrating an example of the circumflex aorta anatomic variant, a rare aortic arch anomaly in which the aorta crosses to the contralateral side posterior to the esophagus and airway resulting in aerodigestive compression.

**Figure 3:**
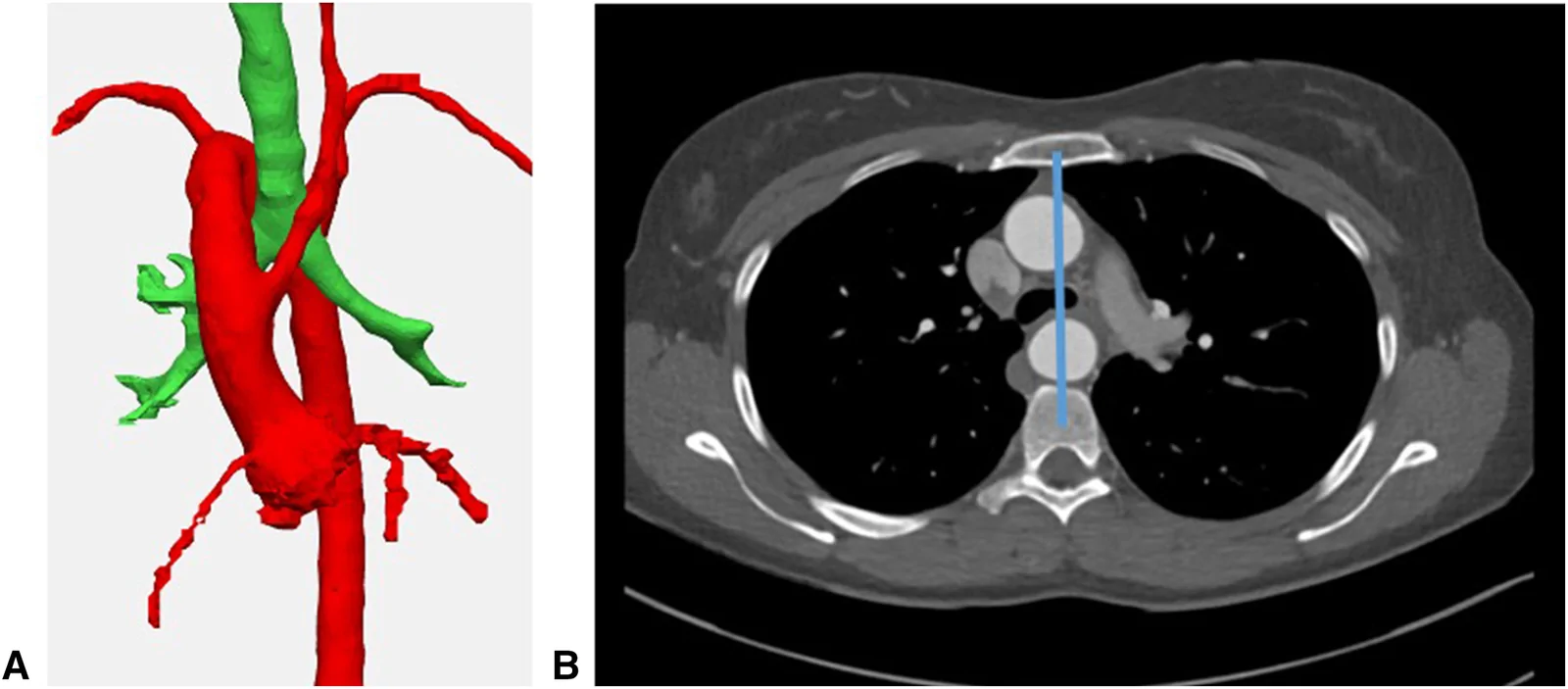
Midline descending aorta: (A) 3D reconstruction demonstrating midline-location of the descending aorta; (B) axial image from a preoperative CT scan, demonstrating midline-location of the descending aorta (*blue line signifying midline*) and resultant aerodigestive compression.

**Figure 4:**
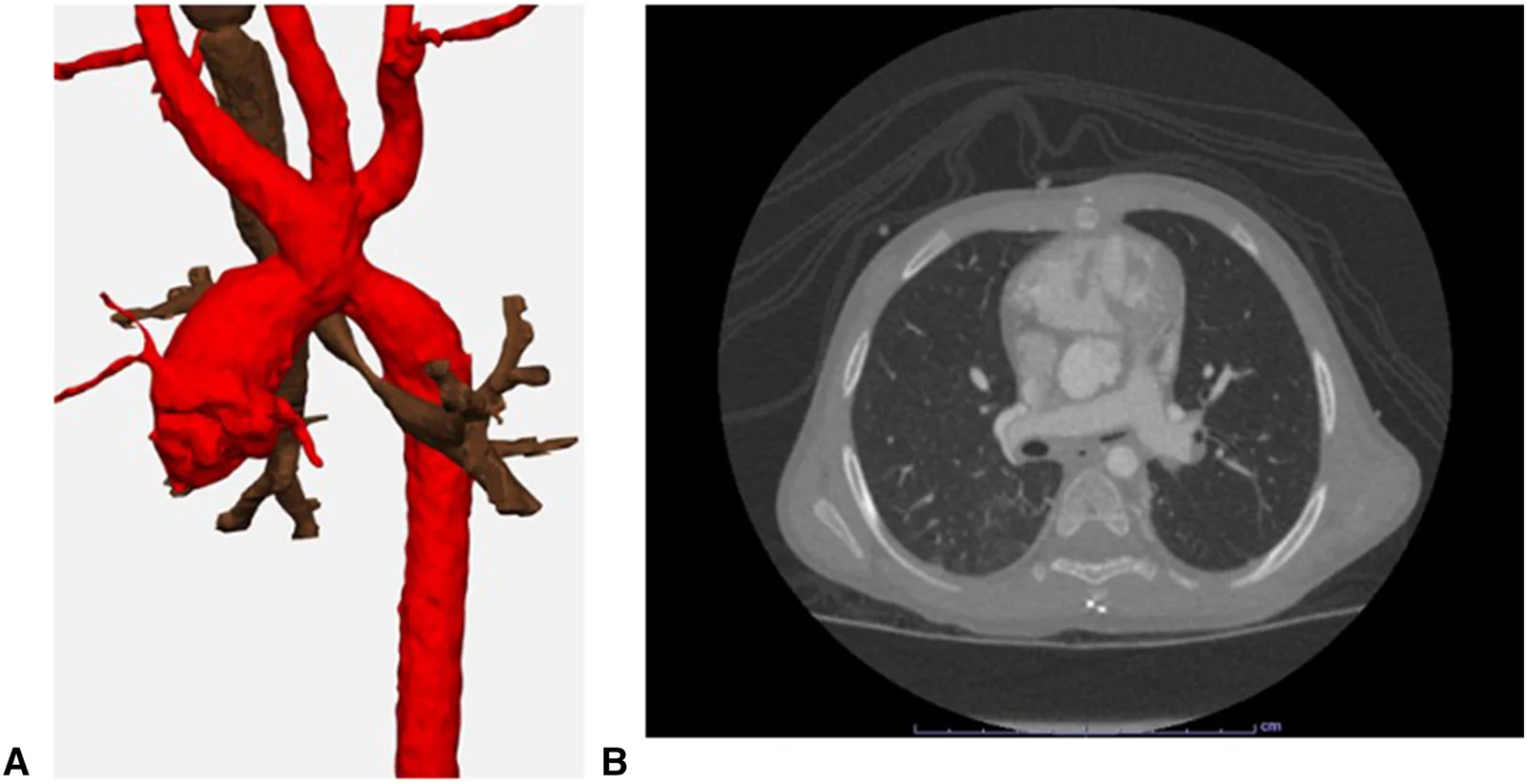
Bowstring aorta: (A) 3D reconstructed model demonstrating the phenomenon of “bowstring aorta,” whereby there is limited space underneath the reconstructed aortic arch (ie from prior aortic reconstructive surgery) that may cause extrinsic pressure on the aerodigestive tract, with corresponding axial image from CT scan (B).

With respect to respiratory symptom evaluation, 3-phase dynamic tracheobronchoscopy and laryngoscopy for vocal fold movement assessment were performed. Three-phase dynamic tracheobronchoscopy was the primary tool used to determine the nature of tracheobronchial compression, distinguishing between dynamic airway collapse related to tracheobronchomalacia and fixed obstruction related to vascular compression.^17^ This approach has been favored by our group over dynamic airway CT, because the bronchoscopic findings may dictate the nature of the tracheobronchial intervention. Vocal fold movement assessment was a critical element of the preoperative evaluation given the potential for preexisting recurrent laryngeal nerve injury incurred during prior vascular ring or aortic arch reconstruction surgeries, as well as the risk of recurrent laryngeal nerve injury associated with undergoing revision vascular ring surgery.^15^^,^^18^

Esophagram and flexible esophagogastroduodenoscopy were used to structurally evaluate the esophagus, particularly to rule out other sources of dysphagia symptoms, such as eosinophilic esophagitis.^15^ Additional functional evaluation with esophageal transit scintigraphy or manometry was also considered on an as-needed basis.

### Operative Technique

Through a median sternotomy, the aortic arch, descending thoracic aorta, trachea, and esophagus were mobilized, including division of the ductal ligament. The right pulmonary artery was mobilized distally to the level of the segmental vessels. Cardiopulmonary bypass was established with aortic and single venous cannulation, and patients were cooled systemically to between 28 °C and 32 °C.

The mid-descending thoracic aorta and distal arch were crossclamped, initiating regional perfusion to the head and heart. The distal arch was transected beyond the subclavian artery, and this site was oversewn. During this time, lower body perfusion was typically performed with an auxiliary pump Y'd off the arterial limb of the cardiopulmonary bypass machine, although in some cases, a separate pump was used for lower body perfusion to control flow. For this, we have preferred to use a sterile endotracheal tube, the size of which was based on the diameter of the descending thoracic aorta. The proximal descending thoracic aorta was then relocated beneath the carina and right pulmonary artery with extreme caution to avoid bilateral recurrent laryngeal nerve injury as monitored with nerve integrity monitoring (see below). After aortic crossclamping and cardioplegic arrest, a transverse aortotomy beginning approximately 1 cm above the sinotubular junction was performed on the ascending aorta, and a posterior longitudinal aortotomy was performed to construct an end-to-side anastomosis of the proximal descending aorta to the posterior aspect of the ascending aorta (Figure 5). Intraoperative flexible bronchoscopy was performed to assess the airway after aortic relocation, with additional anterior/posterior tracheopexy or bronchial splinting performed as needed.

**Figure 5:**
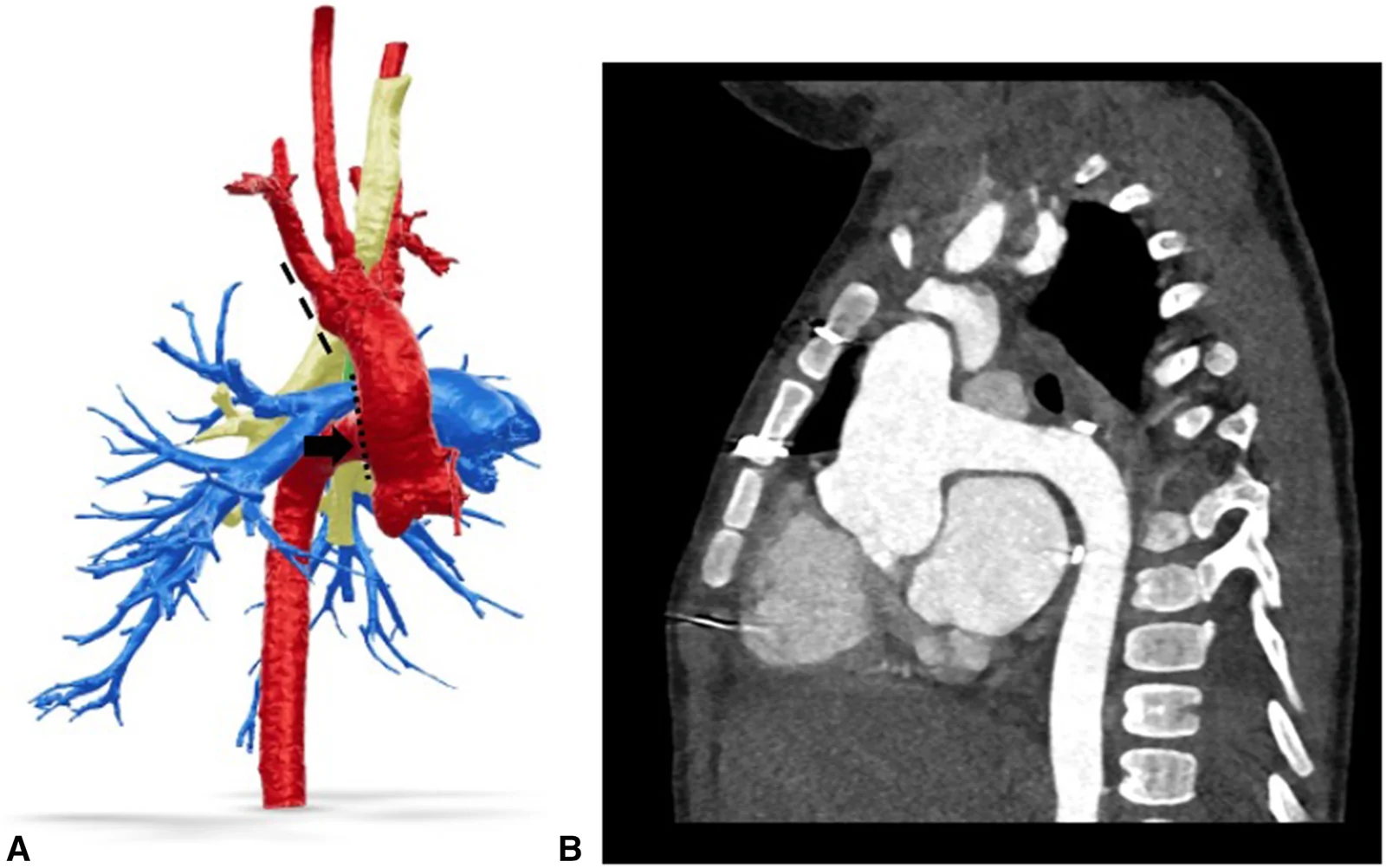
Descending aortic translocation: (A) 3D model demonstrating postoperative configuration after descending aortic translocation, with end-to-side anastomosis of the proximal descending aorta (location of division signified by heavy *dashed line*) to the posterior aspect of the ascending aorta (location of new anastomosis signified by *black arrow/dotted line*); (B) additional image captured from a postoperative CT scan demonstrating the proximal descending arising from the ascending aorta.

Of note, extensive mobilization of the esophagus is often required to relieve the esophageal compression, including with division of fibrous bands at the area of esophageal compression, and esophageal rotation to move the esophagus away from the aorta. Careful attention is paid to the final position of the esophagus with respect to the relocated aorta, with a general preference by our group to keep the esophagus positioned to the patient's left side. However, the preoperative position—particularly the cervical portion—must be considered to avoid crossing the midline afterwards.

### Intraoperative Recurrent Laryngeal Nerve Monitoring

Intraoperative recurrent laryngeal nerve monitoring was performed in all cases using the Nerve Integrity Monitoring (Medtronic) integrated electrode endotracheal tube or a custom-made device using the Dragonfly (Neurovision Medical Products) electrode on a standard endotracheal tube that was placed at the beginning of the case. Notably during the initial dissection, the patients were not muscle-relaxed to facilitate the intraoperative recurrent laryngeal nerve monitoring. After identification of the vagus nerve and the respective recurrent laryngeal nerve branches and completion of the posterior mediastinal and aortic arch mobilization, paralytic was administered.

### Statistical Methods

Continuous variables are presented as median with range or mean with SD. Categorical variables are presented as absolute counts with percentages. The statistical analysis was conducted in R 4.3.3 (R Foundation for Statistical Computing).

## Results

From 2020 to 2023, there were 15 patients with complex aortic anomalies who underwent descending aortic translocation at our institution. Anatomic variants included hairpin aorta (n = 2, 13%), circumflex aorta (n = 4, 27%), midline descending aorta (n = 7, 47%), and bowstring aorta (n = 2, 13%). Although most patients (9/15, 60%) had previously undergone at least 1 prior cardiac operation—such as ligamentum ligation/division, subclavian reimplantation, and aortopexy—to address preexisting congenital vascular ring anomalies, the descending aortic translocation procedure served as the index operation for 6 of 15 patients (40%). Median age was 2 years (range, 8 months to 43 years) (Table 1).

**Table 1:** Patient characteristics

Patient characteristics and outcomes	
Total no. of patients	15
Age, y (median, range)	2 (0.7-43)
Underwent previous vascular ring operations, n (%)	9 (60%)
Presenting symptoms, n (%)	
Respiratory only	2 (13%)
Dysphagia only	1 (7%)
Mixed	12 (80%)
Anatomy, n (%)	
Hairpin aorta	2 (13%)
Circumflex aorta	4 (27%)
Midline descending aorta	7 (47%)
Bowstring aorta	2 (13%)
Cardiopulmonary bypass time, min (mean, SD)	115 (46)
Operative time, min (mean, SD)	477 (96)
Complications, n (%)	
Mortality	0 (0%)
Stroke	1 (7%)
Persistent vocal cord dysfunction	2 (13%)
Chylothorax	1 (7%)
Persistent tachycardia	3 (20%)
Need for reintervention (open, catheter-based, or endoscopic)	3 (20%)
Follow-up time, mo (mean, range)	16.5 (2-31.5)
Relief of symptoms on follow-up, n (%)	
Respiratory	13 (93%)
Dysphagia	9 (69%)
Neither	1 (7%)

Preoperatively, all 15 patients were severely symptomatic from aerodigestive vascular compression, with esophageal symptoms characterized by dysphagia (to solids or liquids) and airway symptoms marked by chronic cough/shortness of breath, desaturation episodes requiring nasal cannula oxygen supplementation, and recurrent upper respiratory infections. Thirteen patients (87%) had mixed esophageal and airway symptoms, 1 patient (7%) had respiratory symptoms only, and 1 patient (7%) had only dysphagia. Notably, 3 patients had preexisting vocal fold paralysis at the time of evaluation for descending aortic translocation as a result of prior operations, with all 3 showing evidence of left vocal fold immobility on preoperative laryngoscopy.

Median cardiopulmonary bypass time was 100 minutes (range, 59-197 minutes). Median aortic crossclamp time was 43 minutes (range, 25-140 minutes). At the time of the descending aortic translocation, tracheopexy (n = 10) or external bronchial splinting (n = 4) was also performed to address concomitant tracheobronchomalacia. Rotational esophagoplasty was performed in more than half of cases (n = 8) to both accommodate posterior tracheobronchopexy and release the fibrous bands entrapping the esophagus for decompression (Table 2). Intraoperative bronchoscopy at the completion of the operation revealed improved airway patency for all 15 patients.

**Table 2:** Detailed patient data

Patient	Age (y)	Original anatomy	Descending aorta position	Previous operation(s)	CPB time (min)	CCT (min)	Concomitant decompressive airway procedure	Concomitant decompressive esophageal procedure
1	2	Right arch/aberrant left (hairpin aorta)	Right	Ligation and division of ligamentum, subclavian to carotid transposition	144	32	Anterior/posterior tracheopexy, Right bronchial splint	Leftward rotational esophagoplasty
2	11	Right arch/MIB (hairpin aorta)	Right	Posterior aortopexy ×2, anterior aortopexy ×2, anterior tracheopexy ×2, posterior tracheopexy, bilateral bronchopexy, right pulmonary artery-pexy	122	27	Anterior tracheopexy, Right bronchial splint	None
3	0.8	Left arch/aberrant right (circumflex aorta)	Right	None	66	25	Anterior/posterior tracheopexy, Posterior right mainstem bronchopexy	Leftward rotational esophagoplasty
4	7	Right arch/aberrant left	Midline	Ligation/division of ligamentum, aortopexy, reimplantation of L subclavian, oversewing of diverticulum of Kommerell	102	29	Posterior tracheopexy, Posterior right mainstem bronchopexy	None
5	43	Double aortic arch	Midline	None	193	53	Posterior tracheopexy	Esophageal mobilization
6	0.8	Right arch/aberrant left (circumflex aorta)	Left	None	138	105	None	Leftward rotational esophagoplasty
7	5	Right arch/aberrant left	Midline	None	77	35	Posterior tracheopexy, Right mainstem bronchopexy	Leftward rotational esophagoplasty
8	1	Hairpin aorta	Left	Posterior aortopexy	186	48	Left bronchial splint	Limited esophageal mobilization
9	0.7	Hairpin aorta	Midline	Cervical tracheopexy	77	43	Left bronchial splint	None
10	0.9	Left arch/aberrant right (circumflex aorta)	Right	None	95	44	Posterior tracheopexy	Leftward esophageal mobilization
11	1	Left arch/aberrant right (circumflex aorta)	Right	None	86	65	Posterior tracheopexy	Leftward rotational esophagoplasty
12	2	Double aortic arch	Midline	Double aortic arch division	100	45	Posterior tracheopexy, posterior R mainstem bronchopexy	Rightward rotational esophagoplasty
13	5	Double aortic arch	Midline	Double aortic arch division	59	33	Posterior tracheopexy	Leftward rotational esophagoplasty
14	5	Right arch/aberrant left	Midline	Ligation/division of ligamentum	83	35	Tracheobronchopexy, posterior R mainstem bronchopexy	Leftward rotational esophagoplasty
15	7	Aortopulmonary window	Left	AP window repair and aortic arch augmentation with pulmonary homograft	197	140	L mainstem bronchial resection with slide bronchoplasty	None

*CPB*, Cardiopulmonary bypass; *CCT*, aortic crossclamp time; *MIB*, mirror image branching; *R*, right; *L*, left; *AP*, aortopulmonary.

Mean follow-up time was 20.1 months (range, 2-32 months). There was no mortality perioperatively or in follow-up. This resulted in 14 of the 15 total patients (93%) experiencing distinct symptomatic improvement. Of the 14 patients with respiratory symptoms, 93% (13/14) experienced symptomatic relief, with 50% (7/14) noting full resolution of prior symptomatology (including frequent respiratory infections, chronic cough, activity intolerance), and 43% (6/14) noting at least partial improvement in symptoms on postoperative follow-up. Of the 14 patients with esophageal compression symptoms, 79% (11/14) experienced improved dysphagia, with 50% (7/14) experiencing full resolution of prior symptoms (including gagging, reflux, and food impaction episodes) and 29% (4/14) experiencing at least partial improvement in oral food tolerance on postoperative follow-up. One patient had neither improvement in respiratory nor esophageal symptoms. Postoperatively, the standard practice of our institution includes obtaining a CT scan to establish a new anatomic baseline and rule out further complications, such as pulmonary artery compression.

Postoperative complications included a neurologic event (n = 1), right pulmonary artery compression requiring stenting (n = 1), chylothorax (n = 1), persistent tachycardia (n = 3), and need for reoperation (n = 2). The neurologic event was a perioperative cardioembolic stroke resulting in temporary hemiplegia that occurred in the 1 adult-aged patient. The patient required discharge to a rehabilitation facility but had full recovery of mobility and functionality, and full symptomatic improvement in preoperative aerodigestive compression symptoms. With regard to the 3 of 15 patients (20%) who experienced persistent postoperative sinus tachycardia after the operation, this may have been due to the extensive posterior mediastinal dissection. With respect to reoperation, 1 patient required delayed redo tracheobronchopexy for recurrent tracheobronchomalacia performed via thoracotomy, and 1 patient required additional esophageal mobilization in the early postoperative period for persistent dysphagia after failed endoscopic esophageal dilation. In both cases, symptoms completely resolved thereafter. Among the 3 patients with known preoperative vocal fold dysfunction, they were evaluated and co-managed postoperatively by the otolaryngology team and ultimately did not require changes to standard perioperative/postoperative care. All 3 patients had noted improvement in aerodigestive compression symptoms postoperatively, without evidence of worsening vocal fold dysfunction Three patients were found to have new unilateral vocal fold dysfunction on postoperative evaluation (left vocal cord hypomobility or paralysis), with complete resolution in the 1 patient with vocal fold hypomobility during follow-up; in the remaining 2 patients (both with left vocal fold paralysis), both were managed with long-term dietary adjustment to thickened liquids given persistent left vocal cord dysmotility, with ongoing surveillance.

## Discussion

The course of the aorta as it relates to the airway and esophagus is an important potential source of aerodigestive compressive symptoms. In this case series, we present a total of 15 patients who underwent descending aortic translocation at our institution over a 3-year period (2020-2023). We demonstrate that this operative approach is feasible and effective, with 14 of 15 patients (93%) achieving at least partial improvement on postoperative follow-up in respiratory or esophageal compression symptoms. For this particularly challenging set of patients, the majority of whom had already undergone a prior vascular ring repair to address aerodigestive vascular compression, descending aortic translocation was effective at relieving persistent airway and swallowing symptoms. By mobilizing and reanastomosing the malpositioned descending aorta in a more favorable configuration with regard to the airway, this allowed for complete offloading and relief of the compressive effects. Moreover, by performing aortic crossclamping distal to the head vessels, this maintains regional perfusion and avoids full circulatory arrest to the brain, thereby limiting—although perhaps unable to fully prevent, given 1 instance of a perioperative neurologic event—neurologic sequelae of this major aortic arch reconstruction.

It is also notable that although several patients had preexisting vocal fold paralysis related to prior operations, the descending aortic translocation operation not only was still successful in providing symptomatic improvement of aerodigestive compression but also did not induce worsening of the vocal cord dysfunction. This supports the safety and feasibility of this procedure even among those with preexisting vocal fold injury or impairment.

Despite the low morbidity/mortality observed in this cohort, careful selection of appropriate candidates for this approach remains a challenge. Additionally, the full ramifications of this extensive operation remain to be seen. A subset of patients (3/15, 20%) experienced persistently elevated resting heart rates—a phenomenon we have also observed after other posterior mediastinal operations—with associated symptomatology such as dizziness. Of these patients, 1 experienced improvement over time, with the ability to return to recreational sports. Although the exact etiology remains unclear—as well as particularly challenging to parse out as patients transition their long-term outpatient follow-up care to outside hospitals closer to home—one possible explanation is that the division of parasympathetic fibers from extensive posterior mediastinal dissection may be a contributing factor. Further follow-up and workup, such as with longer-term heart-rate monitoring via Holter monitors, could be considered and will be further considered.

As our group has previously published, common factors contributing to the need for reoperation after vascular ring repair include ongoing vascular compression, development of constricting scar tissue reforming the vascular ring, tracheobronchomalacia, or a combination.^15^ Several studies have examined the persistence of aerodigestive symptoms after standard vascular ring repair, although the proportion of patients with residual symptoms has varied greatly in the literature.^19^ Whereas Yu and colleagues^20^^,^^21^ have reported persistent postoperative aerodigestive symptoms in as low as 5.6% of patients, Alsenaidi and colleagues^21^ have reported an incidence as high as 54% with persistent respiratory symptoms. Persistent respiratory symptoms appear to be generally more common after vascular ring repair, with several groups reporting symptoms of recurrent respiratory infections, persistent cough, and stridor in 19% to 43% of cases.^22^, ^23^, ^24^, ^25^, ^26^ On the other hand, persistent gastrointestinal or dysphagia symptoms ranged from 6% to 28%.^21^^,^^23^, ^24^, ^25^, ^26^, ^27^, ^28^ Despite the frequency of reported persistent symptoms, these figures may in fact be an underestimate, given the potential for physicians to attribute postoperative symptoms to alternative diagnoses, particularly in the setting of pervasive beliefs that simple relief of a vascular ring should be sufficient to resolve related symptomatology.

Notably, the overall contribution of aerodigestive compression related to aortic position and relative mediastinal space to the phenomenon of postoperative persistence of symptoms is not well understood, given the unknown frequency of these lesions in vascular ring patients and in the general population. Moreover, the degree to which these lesions are pathologic and cause clinically significant symptomatology is also not well understood in the general population. Although our series includes several patients for whom the descending aortic translocation procedure represents the index operation, it is typically considered a second-line procedure, because the role of descending aortic translocation as a primary operation in patients with vascular rings remains uncertain given the increase in risk associated with cardiopulmonary bypass with regional cerebral perfusion in comparison with more standard vascular ring approaches. Ultimately, however, the decision to pursue descending aortic translocation, whether as first- or second-line, is dependent on the anatomic configuration at hand. Although aortic uncrossing may be useful for those with circumflex aorta, in other anatomic configurations (eg, hairpin aorta, bowstring aorta, or midline descending aorta) aortic uncrossing is not feasible, and aortopexy may not adequately address the aerodigestive vascular compression. In these circumstances, the descending aortic translocation is a versatile procedure that allows for increased flexibility to maximally relieve the aerodigestive compression related to these aberrant aortic configurations.

Decision-making is simpler for patients with persistent symptoms after prior vascular ring repair with ongoing evidence of aerodigestive compression. Among patients undergoing repeat vascular ring repair, the descending aorta frequently needs to be addressed.^18^ Despite prior success with descending aortopexy, we have observed that in the reoperative setting, aortopexy alone may not effectively increase the space underneath the aortic arch. In these instances, descending aortic translocation becomes a valuable tool, because the physical relocation of the descending aorta provides an opportunity to fully decompress the aerodigestive tract.

Tracheobronchial compression has been described after aortic reconstruction, with the “bowstring aorta” effect manifesting often as left mainstem bronchial compression after aortic arch reconstruction. Typically, this phenomenon is the result of inadequate mobilization of the descending aorta, an overly patulous arch patch, or very proximal implantation of the proximal descending aorta into the distal ascending/proximal aortic arch when using a direct end-to-side technique. Anterior and posterior tracheopexy have been described to address this issue of tracheobronchial compression.^1^, ^2^, ^3^, ^4^^,^^29^ However, in these patients, aortopexy is ineffective at best and at worst may exacerbate the bowstring effect, because it fails to address the underlying issue of inadequate space underneath the aortic arch. As such, the options for resolving these compressive symptoms are ascending aortic elongation, descending aortic elongation,^5^ or descending aortic translocation. Our general preference is to avoid using interposition grafts for aortic elongation in children given the ongoing need for somatic growth.

Patients with circumflex aorta—a rare congenital aortic arch anomaly characterized by retroesophageal crossing of the aorta to the contralateral side—pose a unique surgical challenge. Both aortic uncrossing and descending aortic translocation are viable and feasible treatment options; however, there is limited available evidence favoring one technique over the other. The choice between these approaches may depend on a variety of factors, including surgeon experience, the degree of mainstem bronchial compression caused by the descending aorta, the midline position of the descending aorta, and the anatomic positioning of the aortic crossing. Specifically, when the location of the aortic crossing occurs at or above the mid-trachea, aortic uncrossing may be preferred, whereas crossings located below the mid-trachea or carina may be better addressed with aortic relocation. It is also noteworthy that patch material is frequently necessary among patients undergoing aortic uncrossing, although the significance of this remains uncertain. With aortic translocation, it is unclear whether the anatomic alterations may ultimately result in chronic changes in blood flow to the lower body, and the long-term effects are yet to be determined.

### Limitations

This was a retrospective single-center cohort study from a quaternary care center, and thus there are distinct limitations that exist. The patients in this series represent a select group of patients who were referred to our center; thus, the true denominator of patients with these aortic configurations is unknown. Additionally, there was no comparator group undergoing alternative therapies for aerodigestive compression. Finally, the short duration of follow-up means that the overall durability of symptomatic relief remains unknown.

## Conclusions

Descending aortic translocation is a novel operative approach that can provide symptomatic relief for patients with aerodigestive compression related to aortic malposition. Despite the effectiveness of this operation in a variety of settings, the potential for morbidity is greater for this approach compared with more standard vascular ring repair techniques. For this reason, the role of this operation as first-line therapy for patients who present with vascular rings and complex aortic anatomy remains uncertain. However, as the experience with this operation continues to grow, it may be possible to identify a subset of patients at enhanced risk for reoperation who would thus benefit from descending aortic translocation as the primary operation.

## Conflict of Interest Statement

The authors reported no conflicts of interest.

The *Journal* policy requires editors and reviewers to disclose conflicts of interest and to decline handling or reviewing manuscripts for which they may have a conflict of interest. The editors and reviewers of this article have no conflicts of interest.
